# Uncertainty Analysis of Fiber Optic Shape Sensing Under Core Failure

**DOI:** 10.3390/s25082353

**Published:** 2025-04-08

**Authors:** Francesco Falcetelli, Leonardo Rossi, Raffaella Di Sante, Gabriele Bolognini

**Affiliations:** 1 Department of Industrial Engineering—DIN, University of Bologna, 47121 Forlì, Italy; francesco.falcetelli@unibo.it (F.F.); raffaella.disante@unibo.it (R.D.S.); 2 Consiglio Nazionale delle Ricerche, 40129 Bologna, Italy

**Keywords:** optical fiber sensors, shape sensing, uncertainty analysis, core failure, structural health monitoring

## Abstract

Shape sensing with optical fiber sensors is an emerging technology with broad applications across various fields. This study evaluates the metrological performance of shape sensing cables in the presence of fiber core failures, a critical issue in scenarios where cable replacement is impractical due to technological and economic constraints. The impact of core failure is quantified by comparing the uncertainty in key parameters, such as curvature and bending angle, between pristine and damaged cables through Monte Carlo simulations. Results indicate that while core failure degrades performance, shape reconstruction remains achievable. However, the reconstruction becomes sensitive to bending direction due to the loss of core symmetry. Additionally, simulations of how measurement noise propagates into uncertainty in the 3D shape reconstruction are carried out. Analysis of specific shapes, including a circle and a right-handed helix, shows that increasing the number of sensing cores significantly mitigates the adverse effects of core failure. The most notable improvement occurs when the number of cores is increased from four to five. These findings show how shape reconstruction is still possible even in the presence of core damage, and how this changes the behavior of the sensing process.

## 1. Introduction

In structural health monitoring (SHM), optical fiber sensors (OFSs) are considered one of the most promising technologies thanks to their ability to provide a non-invasive characterization of strain along large-scale structures with the use of a single interrogating device [1]. Strain data can be utilized to locate damage, and the effectiveness of damage detection can be quantified using probability-of-detection curves [2,3].

**Figure 1:**
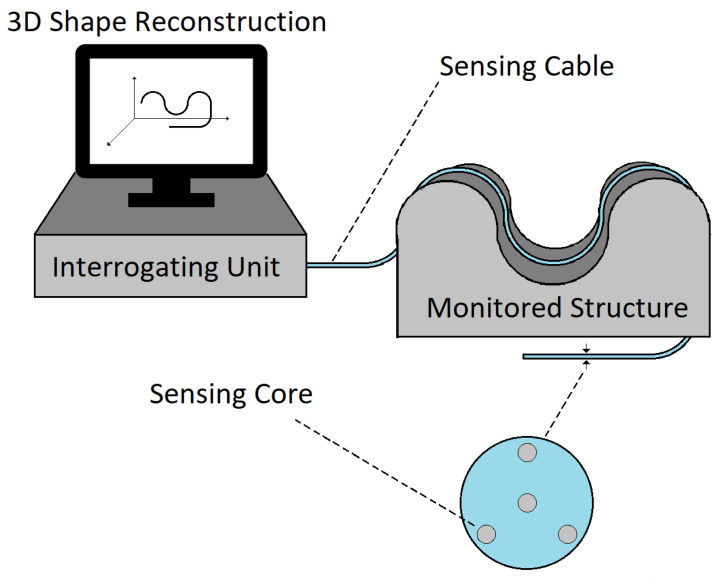
Shape sensing with OFSs.

In recent years, the strain sensing capabilities of OFSs have been adapted for shape detection [4]. Shape sensing is achieved using cables containing multiple single-core optical fibers (fiber bundles) or a unique multi-core optical fiber (Figure 1). From the strain measurements of individual fibers (or individual cores in the same fiber), it is possible to reconstruct the three-dimensional shape of the sensing cable (or fiber) using the Frenet–Serret formulas or equivalent approaches [5].

The strain measurement can be obtained using a quasi-distributed system based on Fiber Bragg Grating (FBG) sensors or by distributed sensing technologies, such as ones based on Rayleigh and Brillouin scattering, which offer the possibility of long measurement ranges and a high number of sensing points. The choice of either sensing cables containing fiber bundles or multi-core optical fiber is closely related to the specific application.

In particular, sensing cables based on multi-core fibers are mostly used in small-scale applications, such as biomedical and robotics [6,7,8,9], where the size and compactness of the sensing cable are of concern.

For applications where size is not a concern, such as SHM for civil infrastructures, sensing cables made from bundles of standard single-mode optical fiber (ITU-T Rec. G.652) can have much greater sensitivity to bending because of the increased distance between their cores and the central axis, which improves sensitivity.

In both cases, the study of uncertainty propagation is of great importance from a metrological point of view, and several authors have investigated it. Henken et al. examined the impact of design parameters on position accuracy in an FBG-based shape sensing model. They considered the accuracy of wavelength measurement, sensor geometry, various sensor configurations, and interpolation models [10]. Floris et al. studied the effect of measurement errors [11] and core position uncertainty [12]. It was discovered that the level of uncertainty in the estimated curvature and bending angle values is directly related to the standard deviation of the measurement noise. Additionally, the uncertainty in the core position also significantly impacts the effectiveness of shape sensing. Megens et al. assessed the shape accuracy of an FBG-based sensing system for medical devices [13]. They conducted bench experiments to determine the accuracy of the tip location and orientation by displacing and rotating the fiber end while allowing the rest of the fiber to bend naturally. The uncertainty in the tip location and orientation was found to be below the millimeter and milliradian levels, respectively. Falcetelli et al. investigated the influence of uncertainty in strain measurements and core positioning, accounting for both random fluctuations and coordinated rotations, on the curvature and bending angle parameters [14]. The latter source of uncertainty can be thought of as the result of a not-perfect spun of the fiber resulting from the manufacturing process.

Another aspect that was taken into account was the bias introduced in the final reconstructed shape by the specific numerical approach used to model shape sensing. This bias can originate from underlying hypotheses or from numerical instability caused by the presence of noisy data or specific shapes. For instance, while the most widely used approach is based on the Frenet–Serret equations [5], other authors have introduced more sophisticated shape sensing algorithms to overcome some of the limitations of this approach [15,16,17,18].

From the literature review, it emerges that despite efforts to assess uncertainty propagation in shape sensing, there are still several aspects that deserve further investigation. One of these aspects is the uncertainty analysis in the presence of core failure—that is, the loss of a sensing core due to damage—which is a real possibility in some applications, where it cannot always be repaired and will significantly affect the functionality and accuracy of the shape sensing process. Sources of damage that can lead to core breakage can occur during the cable installation process, as accidental impacts or damage during the pre-tensioning process can break one of the sensing cores, especially in very long cables where substantial friction must be overcome.

Different levels of mechanical stress on the fiber will surely affect the failure rate of the cores in both installation and operation phases. Antunes et al. characterized the mechanical properties, like the elastic constant, the Young modulus, and the mean strain limit for commercial optical fibers [19]. Leveraging the work of Danzer et al. [20], the authors used a Weibull law (although, in certain cases, deviations from Weibulls statistics can occur) to establish the probability of optical fiber breakage with the applied level of stress. The results show a clear increase in the probability of fiber breakage for higher stress levels. This means that particular attention should be paid to the tension of the shape sensing cable during the installation process. But also, those applications where the curvature (hence the maximum strain levels) are higher (i.e., medical, robotics, and other applications with small bending radii) are the ones exposed to a higher risk. Moreover, according to Danzer et al., the applications where optical fiber failure is most likely to occur are those with long optical fibers, since it is more likely to find a flaw in a larger structure (mainly SHM applications) [20]. Therefore, the problem of core failure is of interest for all shape sensing applications.

This risk is further exacerbated in shape sensing cables deployed in harsh environmental conditions, which can intensify stresses on the cable. Indeed, it is a known fact that aggressive environments can lead to fiber degradation and damage, and that the addition of layers can improve different mechanical characteristics, including the Young modulus, resulting in overall higher resistance [19]. While additional layers, such as cable jackets, provide improved protection and mitigate the effect of harsh environments, they can result in reduced spatial resolution due to the strain transfer mechanism from the outer structure to the fiber core [21]. Another source of core breakage is linked to the cable design: if signal quality was the only concern, the ideal design would involve positioning the sensing cores as far from the neutral axis as possible while minimizing the protective layer. This way, the strain the sensing cores would experience with bending would be maximized, resulting in an optimal signal-to-noise ratio. However, increasing the strain of the sensing cores inherently increases the risk of breakage when compounded with other sources of stress. It should be noted that, due to the nature of bending, each core will experience a different amount of strain, so it is possible that only a single core will experience sufficient stress to break. Aside from mechanical damage, issues with the interrogator can lead to situations equivalent to core failure. In cases where the interrogator can monitor each sensing fiber as a separate channel, a damage in one of the cores would result in that channel being excluded from the measurement. In cases where interrogator technology necessitates the use of a single channel to interrogate multiple cores, the cable design involves a fiber running back and forth with connection splices at the cable ends. Despite protective measures, these splices can fail due to their inherent fragility, thereby rendering the reading of certain cable cores impossible. In the context of very long shape sensing cables, such as those used in civil engineering applications, the likelihood of manufacturing defects increases. These defects can lead to localized stress concentrations, causing core failure. In addition, repeated fatigue cycles, driven by substantial temperature variations, can also contribute to core degradation, eventually resulting in failure.

To the best of the authors’ knowledge, uncertainty analysis in the presence of core failure has never been investigated. The occurrence of core failure is extremely important to be addressed in a decision-making framework. Indeed, the engineer needs a tool to evaluate whether the sensing cable is still able to reconstruct the shape within a certain level of uncertainty or, alternatively, whether it should be replaced to ensure the reliability of the monitoring system. In addition, the cost and difficulty of replacement must also be taken into account. For example, Chanda et al. provide a mathematical framework for assessing the cost savings associated with the installation of an SHM system considering all the expenses associated with the structure lifecycle [22]. A similar approach could be used to provide a decision-making framework for shape sensing applications, in particular in case of damage.

Furthermore, uncertainty is typically assessed in relation to curvature and bending angle values for a cross-section in the sensing cable, without considering how uncertainty spreads in three-dimensional space.

In this work, we use Monte Carlo simulations to assess the metrological performance of different shape sensing cables and how it changes when a single core breaks, both in terms of point-like measurement of the curvature vector (defined by the two values of curvature magnitude and bending direction) and in terms of full 3D reconstruction, using two examples of simple shapes. Through these simulations, we analyze how the loss of a sensing core makes the measurement asymmetrical, with measurement uncertainty becoming dependent on the curvature vector, and how this affects the overall performance, which is an important factor for industrial applications, where the probability of detection for specific events always needs to be properly defined [3]. By simulating sensing cables with different numbers of cores, we were also able to shed light on how resilience to core breakage improved with more sensing cores.

The simulations performed in this work are intended to be independent of the specific optical fiber sensor used to obtain strain data. As a consequence, while we mainly took into consideration sensors based on single-mode fibers, since these are by far the most common fiber types employed by distributed strain sensor systems used for shape reconstruction, the results are independent of the specific optical fiber sensor and can also be applied to sensing systems employing different types of sensing fibers.

There are three potential points of novelty for this work: first, this is the first paper that considers the possibility of measurement in the presence of core failure and how it can still be performed. Second, this work reveals non-trivial results indicating that the loss of symmetry in the sensing cable results in a measurement accuracy that is asymmetrical with respect to the bending direction, and the nature of this asymmetry, which has implications in practical applications where the performance of a broken sensing core must be characterized, especially for shapes lying on a plane (i.e., with a preferential bending direction). Third, we were able to provide guidelines for the sensor design, indicating a specific cable configuration (four outer cores + one central core) as the best compromise between low accuracy loss from core breakage and low design complexity.

## 2. Materials and Methods

### 2.1. Shape Sensing Theory

**Figure 2:**
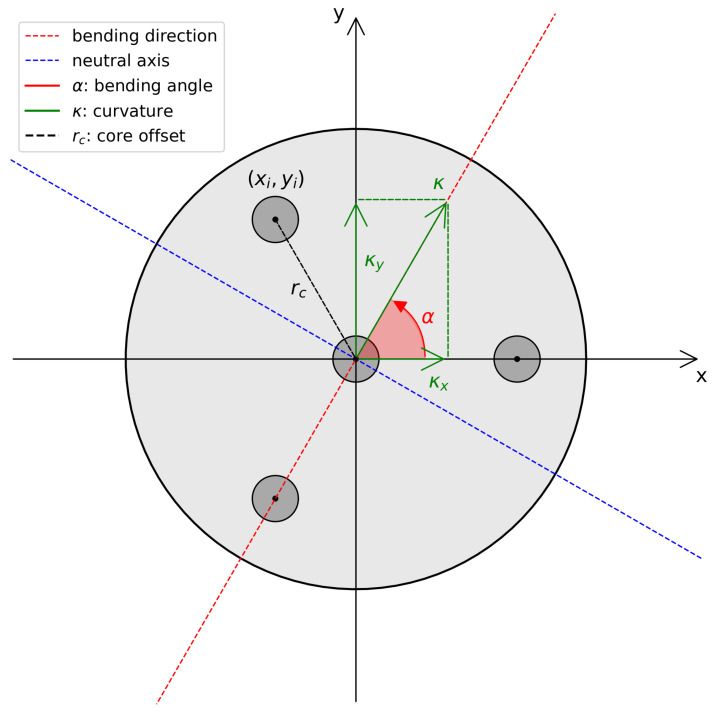
Shape sensing cable cross-section.

In general terms, shape sensing through optical fiber sensors is made possible by the measurement of strain levels of cores that are symmetrically distributed around the main axis of the sensing cable. Being off-axis, these cores will experience different strain levels when the sensing cable bends in a certain direction. At every point of the sensing cable, the situation can be described by a diagram like the one shown in Figure 2.

The bending is represented by the curvature vector κ, which is defined by its modulus κ and the angle α with respect to the reference frame. In this situation, a certain fiber core centered at coordinates $\left(x_{i},y_{i}\right)$ with respect to the center of the cable will experience a strain due to bending equal to

(1) $$\varepsilon_{i}=\varepsilon_{long}+\kappa_{x}x_{i}+\kappa_{y}y_{i}$$

where $\kappa_{x}$ and $\kappa_{y}$ are the *x* and *y* components of the curvature vector, and $\varepsilon_{long}$ is the longitudinal strain.

Therefore, by applying Equation (1) to each fiber core, one obtains a system of *n* algebraic equations, where *n* is the number of cores. The system can be solved for the variables $\varepsilon_{long}$, $\kappa_{x}$, and $\kappa_{y}$, if there are at least three non-aligned cores in the cross-section. When three cores are present, the system is determinate with a unique solution. If more cores, and hence more equations, are present, the system is overdetermined. In this last case, the solution is to solve the problem by minimizing the Sum of Squared Errors (SSE) [12], which can be computed as shown in Equation (2):

(2) $$SSE\left(\varepsilon_{long},\kappa_{x},\kappa_{y}\right)=\sum_{i=1}^{n}{\left(\varepsilon_{i}-\varepsilon_{long}-\kappa_{x}x_{i}-\kappa_{y}y_{i}\right)}^{2}$$

which is equivalent to nullifying the gradient, as follows:

(3) $$\nabla SSE\left(\varepsilon_{long},\kappa_{x},\kappa_{y}\right)=0.$$

This condition is equivalent to the following system of equations:

(4) $$\left\{\begin{array}{c} \frac{\partial SSE}{\partial \varepsilon_{long}}=0⟶n\varepsilon_{long}+\kappa_{x}\sum_{i=1}^{n}x_{i}+\kappa_{y}\sum_{i=1}^{n}y_{i}=\sum_{i=1}^{n}\varepsilon_{i}\\ \frac{\partial SSE}{\partial \kappa_{x}}=0⟶\varepsilon_{long}\sum_{i=1}^{n}x_{i}+\kappa_{x}\sum_{i=1}^{n}x_{i}^{2}+\kappa_{y}\sum_{i=1}^{n}x_{i}y_{i}=\sum_{i=1}^{n}x_{i}\varepsilon_{i}\\ \frac{\partial SSE}{\partial \kappa_{y}}=0⟶\varepsilon_{long}\sum_{i=1}^{n}y_{i}+\kappa_{x}\sum_{i=1}^{n}x_{i}y_{i}+\kappa_{y}\sum_{i=1}^{n}y_{i}^{2}=\sum_{i=1}^{n}y_{i}\varepsilon_{i} \end{array}\right.$$

In the case where the fiber cores are symmetrically distributed, the system is simplified into the form:

(5) $$\left\{\begin{array}{c} n\varepsilon_{long}=\sum_{i=1}^{n}\varepsilon_{i}\\ \kappa_{x}\sum_{i=1}^{n}x_{i}^{2}=\sum_{i=1}^{n}x_{i}\varepsilon_{i}\\ \kappa_{y}\sum_{i=1}^{n}y_{i}^{2}=\sum_{i=1}^{n}y_{i}\varepsilon_{i} \end{array}\right.$$

The system of equations, when solved, will provide the solutions for $\varepsilon_{long}$, $\kappa_{x}$, and $\kappa_{y}$. From these values, it is then trivial to extract the modulus and angle of the curvature vector:

(6) $$\kappa =\sqrt{\kappa_{x}^{2}+\kappa_{y}^{2}}\;\;\;\;\mathrm{and}\;\;\;\;\alpha =\mathrm{angle}\left(\kappa \right)$$

The three-dimensional shape of the cable can be described as a parametric curve $\gamma \left(s\right):\left[0,L\right]⟶R^{3}$, where *L* is the total length of the sensing cable. Thus, each sensing point *i* of the cable at distance $s_{i}$ from the origin will correspond to a point of the curve, with position $\gamma (s_{i}),\;i=0,\ldots ,N$ in the three-dimensional space.

In order to extract the 3D shape of the sensing cable from the curvature vector at each sensing point, different techniques can be used. One of the most widely adapted, and the one we will focus on in this work, is based on the Frenet–Serret frame [23], which is defined by three vectors: the tangent to the curve (T), its derivative with respect to the length parameter of the curve, or normal (N) and their cross product, or binormal (B). The Frenet-Serret formulas can be written as:

(7) $$\left[\begin{array}{c} T^{\prime }\left(s\right)\\ N^{\prime }\left(s\right)\\ B^{\prime }\left(s\right) \end{array}\right]=\left[\begin{array}{ccc} 0 & \kappa \left(s\right) & 0\\ -\kappa \left(s\right) & 0 & \tau \left(s\right)\\ 0 & -\tau \left(s\right) & 0 \end{array}\right]\left[\begin{array}{c} T\left(s\right)\\ N\left(s\right)\\ B\left(s\right) \end{array}\right]$$

where *s* is the curve parameter, and τ is the torsion of the curve, which can be calculated as the derivative of the bending angle of the curvature vector along the curve, or $\frac{d\alpha }{ds}$

(8) $$\tau \left(s\right)=\frac{d\alpha }{ds}.$$

Equation (7) represents a set of linear ordinary differential equations and can be solved using many different numerical methods. Then, the final shape of the sensing cable is obtained by integrating the tangent vector with respect to *s* [5]. Alternative approaches to the classic Frenet–Serret frame are also available [15,16,17,18], but, as mentioned in the introduction, they usually provide benefits only in specific situations where the Frenet–Serret frame could face instability, such as quick changes in the bending angle, which are beyond the scope of this paper.

**Figure 3:**
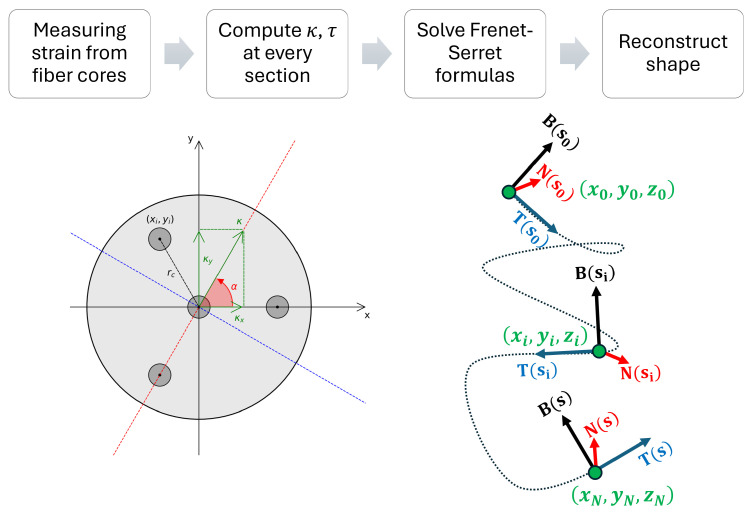
Shape sensing flowchart.

To summarize, the fiber optic shape sensing flowchart is described in Figure 3:

### 2.2. Core Failure Compensation

An aspect that is usually ignored in experiments but is a possibility in actual applications is the eventuality that the strain information in one of the sensing cores becomes unavailable, which can occur for fiber bundles in environments with high degrees of stress, such as the case for SHM. Depending on the application, the fiber bundle might be structured so that all fiber cores are connected together at the beginning and end of the sensing cable so that they form a single fiber interrogated by the sensor, where every segment corresponds to a different core. In this situation, when the fiber in one of the segments breaks, instead of replacing the entire sensing cable, the ends of the core segments can be re-spliced to exclude the damaged section, allowing for the continued use of the sensing fiber at the cost of the loss of a core.

To compensate for the lost core, two different methods can be considered. The first is to use the simplified approach from Equation 5 and set the strain measured by the lost core as the difference between the longitudinal strain (that is, the strain measured in the central core) and the sum of the strain measured in the remaining core: $\varepsilon_{long}-\sum_{i=1}^{n_{cores}-1}\varepsilon_{i}$. Although this value would in principle be equal to the strain that would be measured in the missing core, the estimate would be affected by the combined measurement noise of all the strain measurements from the other cores. Another possibility is to simply ignore the broken core and perform the curvature and bending angle measurement with a now spatially asymmetrical set of cores, using Equation (4).

This process is computationally more expensive, requiring the solution of the system of equations shown in Equation (4), which requires the calculation of the inverse of a 3 × 3 matrix, instead of the calculation of the three formulas in Equation (5). Despite this downside, it can provide a more accurate estimate of the curvature vector and will be the method used in this work.

It is important to note that, in order for the curvature vector to be estimated in all possible directions, at least three non-aligned cores must always be present inside the sensing fiber, meaning that compensation for a missing core can only be performed in sensing cables with at least four cores—either one central core with three outer cores, or four outer cores. We will also assume that the core distribution before breakage will be symmetrical, as is the case for most sensing cables studied in the literature. This is because they maximize the capability of measuring arbitrary curvature vectors while keeping the number of cores constant. In principle, adding multiple cores along a specific axis could help improve the accuracy along a specific bending direction, but these applications usually focus specifically on curvature detection [24].

### 2.3. Design of Simulations

#### 2.3.1. Single-Sensing-Point Simulations

To simulate the effect of measurement noise and single core breakage on the computation of curvature and bending direction, we focus on a single sensing point of the cable. Starting from a set curvature and bending angle, the acquisition is simulated by calculating the strain values of each core using Equation (1) and then adding a noise term equal to a random variable belonging to a normal distribution with a mean of 0 and a standard deviation equal to the measurement noise. The curvature and bending angle are then recalculated using Equation (6). The Monte Carlo simulation is conducted by repeatedly adding noise and recalculating curvature and bending angle for 10^4^ iterations. All the obtained curvature and bending angle values can be sampled statistically, and their standard deviation can be used as the uncertainty of the measurement, while the mean allows us to verify if there are any deviations from the real values.

We decided to simulate measurement noise as Gaussian in a similar way to previous simulation works such as [11]. In this situation, we use this noise to represent all sources of uncertainty that affect the strain measurement process from the optical fiber sensor, which can be considered Gaussian. In addition, since we are focusing on static measurements, we can assume that the acquired strain values are averages over multiple data acquisitions, and thus tend to have a Gaussian distribution due to the central limit theorem.

#### 2.3.2. Full 3D Reconstruction Simulations

To estimate the effect of measurement noise on the shape reconstruction of the entire sensing cable, we start by choosing a curve, which is defined by two vectors, $\kappa_{i}^{real}$ and $\tau_{i}^{real}$, which identify the torsion and curvature value for every sensing point *i*. For simplicity, the original bending angle is set at 0°, and from this, the bending angle at every point *i* can be obtained by integrating $\tau_{i}$. Using Equation (1), it is possible to obtain the real strain values for every fiber core at each sensing point. Then, it is possible to proceed in a way similar to the paragraph above: noise is added to all strain values, and Equation (6) is used to obtain the measured curvature $\kappa_{i}^{meas}$ and bending angle values, from which the torsion $\tau_{i}^{meas}$ is obtained through derivation.

#### 2.3.3. Core Failure Effect on Measurement Symmetry

Since the loss of a core means that the sensing cable is no longer radially symmetrical, the measurement may have different features (e.g., in accuracy) depending on the orientation of the curvature vector. To evaluate this issue, we performed Monte Carlo simulations for a single sensing point in a 4-core (3 outer cores + 1 central core) sensing cable, starting from a real curvature value of 10 m^−1^ and bending angle values ranging from 0 to 360°, with a step of half a degree. Monte Carlo simulations were performed for every orientation of the curvature vector. The simulation parameters are shown in Table 1.

## 3. Results

### 3.1. Core Failure Analysis on a Single Cross-Section

**Figure 4:**
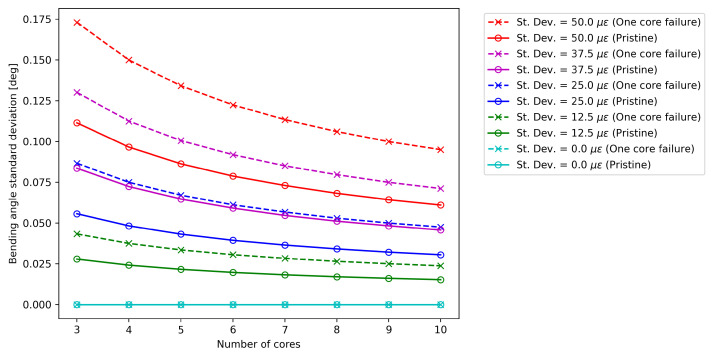
Effect of core number on the bending angle standard deviation for the pristine optical fiber (“o” marks), and in the case of one core failure (“x” marks).

Figure 4 compares the standard deviation of the bending angle $\sigma_{\alpha }$ for the pristine and damaged case at different measurement noise levels for a given cross-section of the sensing cable. In both cases, except for the condition without measurement noise, the standard deviation decreases at a rate proportional to the square root of the number of cores [14]. As expected, the higher the noise level, the higher the value of $\sigma_{\alpha }$. Specifically, it is possible to show that there is a linear relationship between the noise level and $\sigma_{\alpha }$.

**Figure 5:**
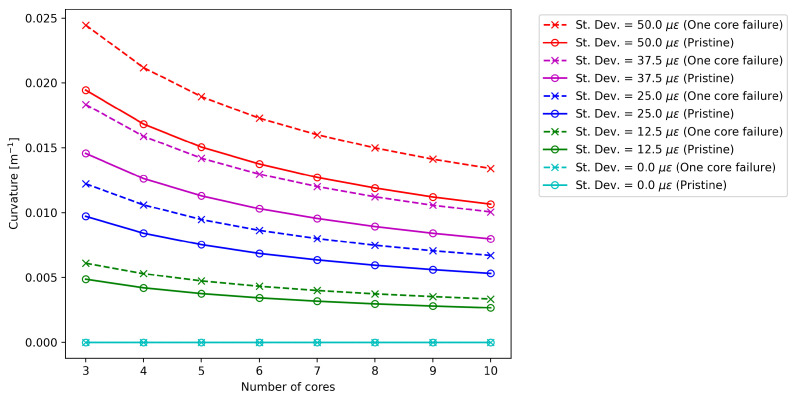
Effect of core number on the curvature for the pristine optical fiber (“o” marks), and in the case of one core failure (“x” marks).

Figure 5 again proposes this type of analysis, but focuses on the standard deviation of the curvature $\sigma_{\kappa }$. The results confirm the beneficial effect of increasing the number of cores in both pristine and damaged cases, with the uncertainty decreasing at a rate proportional to the square root of the number of cores. The data confirm that the uncertainty level increases linearly with the standard deviation of the measured noise imposed in the Monte Carlo simulation.

From a first qualitative analysis, the comparison of Figure 4 and Figure 5 suggests that fiber failure causes a much more severe increase in uncertainty for bending angle compared to curvature: when a core is missing, $\sigma_{\alpha }$ increases by a value ranging from 55% to 56%, while $\sigma_{\kappa }$ increases by a factor ranging from 25% to 26% in the cases taken into account in this work [25]. It is also important to note that, even if the overall noise changes with the number of cores, these factors appear to remain constant.

### 3.2. Polar Analysis

In this section, the results show the sensitivity of the shape sensing cable to the variation in the bending angle in the presence of a damaged fiber. As previously performed, the two key parameters for shape sensing are estimated, namely the bending angle, α, and the curvature, κ, at a given section of the sensing cable.

Monte Carlo simulations are used to evaluate these two parameters in various scenarios. This study focuses on three distinct core number configurations ($n_{c}=4,5,7$), under both pristine conditions and cases involving the failure of a single core in each configuration. The polar coordinates of the failed core are $\left(r_{c},\theta \right)$, where θ is always equal to 30°. Table 1 shows the parameters of the Monte Carlo simulation used in this study.

The core offset, defined as the radial distance of the cores from the center of the cable, was set to 2.1 mm. Although this choice was arbitrary, it does not compromise the generality of the simulation. The value of 2.1 mm was selected because the authors plan to conduct future experimental activities with a shape sensing cable having this specific radial offset. In addition, the literature presents examples of sensing cables with varied core offsets depending on the application, with diameters ranging from fractions of a millimeter, for multi-core fibers, to a few tens of millimeters, for structural health monitoring and geotechnical applications [26,27,28]. The chosen radius value was considered to be representative of the average of sensing cables used for SHM applications, where core breakage is expected to be more prevalent. It is important to note that, for a given curvature vector, the core offset is directly proportional to the detected strain [11], meaning that using a different value only changes the overall signal-to-noise ratio of the measurement and is equivalent to testing different noise levels. Regarding measurement noise, finding a “typical” value is difficult since it depends on a variety of factors, including the specific sensor type and model, as well as how the measurement is performed (for instance, in BOTDA sensors, noise also depends on the number of averages chosen for a specific measurement). As a result, the choice of noise was a guess, but we tested different noise values in Section 3.1, showing how curvature and bending angle measurement showed identical behavior at different noise levels. The measurement noise was set to 50 με, based on available experimental evidence. This value was determined through a trial-and-error procedure to best highlight the outcomes of the results. The choice of the measurement noise is closely related to the radial offset, as both factors influence the signal-to-noise ratio. A higher radial offset enhances the sensitivity of the shape sensing cable, allowing it to tolerate greater levels of noise while maintaining accurate measurements. The target curvature was set to 10 m^−1^, with the assumption that there was no longitudinal deformation. Although other values could be used for similar studies, these parameters were chosen to align with the range of values used by Floris et al. [11,12]. To ensure adequate resolution in the polar plot, the Monte Carlo simulation was repeated every 0.5°, covering the range from 0 to 2π, resulting in a total of 720 simulations. Given the high number of simulations and the qualitative nature of this stage of the study, the number of Monte Carlo simulations was limited to 10^3^. However, the inclusion of 95% confidence intervals effectively quantifies the uncertainty related to the simulations.

**Figure 6:**
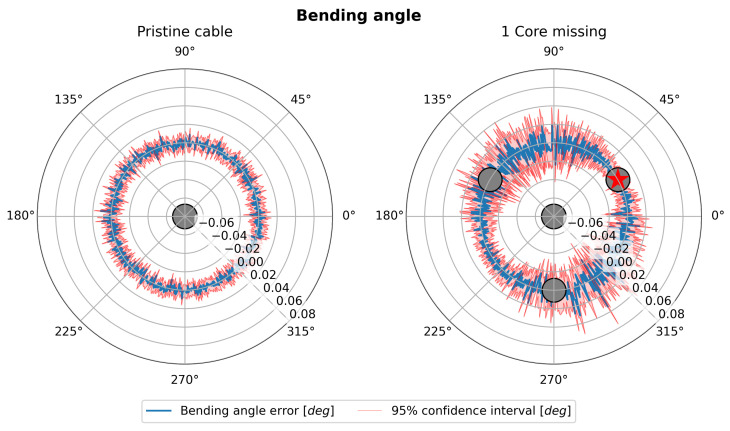
Polar plot of bending angle error.

Figure 6 (left and right) shows the trend of the bending angle error (blue line) as the bending angle itself varies in a polar representation for a shape sensing cable with four cores. The 95% confidence intervals (C.I.) are shown in red. When comparing a perfectly functional sensing cable, as shown in Figure 6 (left), with the case where one core is damaged, as shown in Figure 6 (right), a clear increase in uncertainty in the estimate of the bending angle is evident. Moreover, while the uncertainty in the pristine case has no preferential direction, the symmetry is broken in the damaged case. The uncertainty in estimating the bending angle reaches the maximum when said angle is 90° away from the damaged core. As shown in Figure 2, this corresponds to the broken core lying along the neutral axis of the cable section. This result can be interpreted by considering that the core that is located along the neutral axis of the cable by definition incorporates the information of the bending angle direction and is therefore the most important for its estimation.

**Figure 7:**
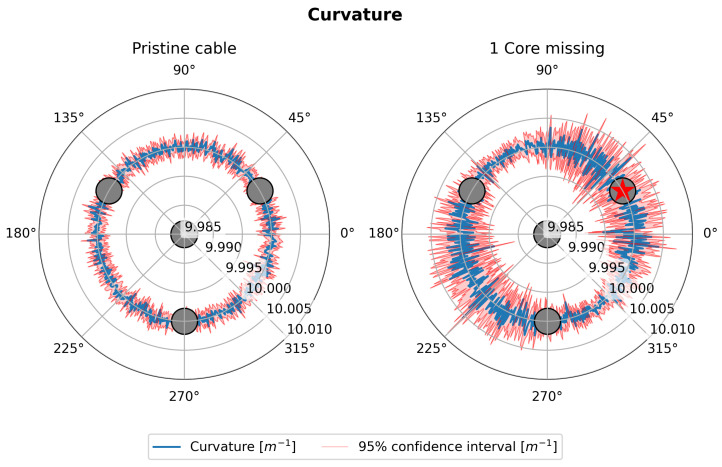
Polar plot of curvature.

Similarly, Figure 7 (left and right) shows the curvature trend (blue line) as the bending angle varies in a polar representation for the same shape sensing cable with four cores. Also, in this case, the 95% C.I. are shown in red. The comparison in the case where the cable is perfectly functional, Figure 7 (left), with the case where one core is damaged, Figure 7 (right), again shows an increase in uncertainty that depends on the value of the bending angle.

However, it is interesting to note that the angles where the uncertainty reaches the maximum are different between the right sides of Figure 6 and Figure 7. In particular, the uncertainty in curvature estimation seems to be at its maximum when the damaged core lies perpendicular to the neutral axis. In this situation, the damaged core would lie exactly where the strain is at its maximum, resulting in the loss of one of the most contributive cores in the estimation of the strain. In contrast, when the damaged core lies on the neutral axis, it would lie in an area where the strain is at its minimum, and the estimation of the curvature would not be affected. The analysis shown by Figure 6 and Figure 7 is repeated, increasing the number of cores.

**Figure 8:**
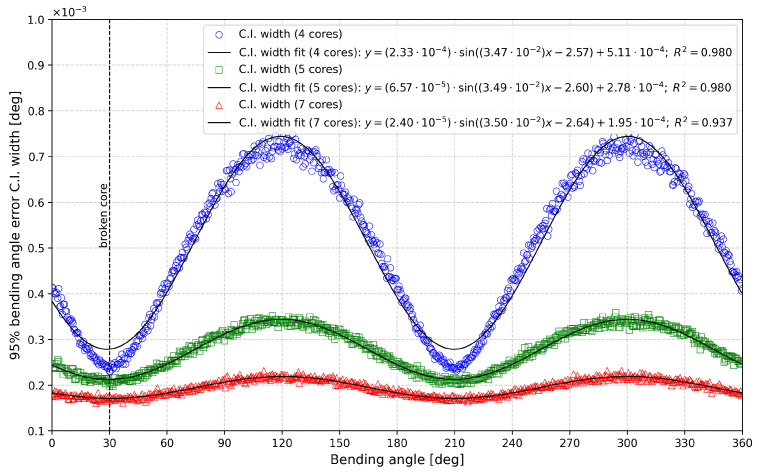
Representation of 95% bending angle error C.I. width as a function of the bending angle for different numbers of cores in the damaged case.

**Figure 9:**
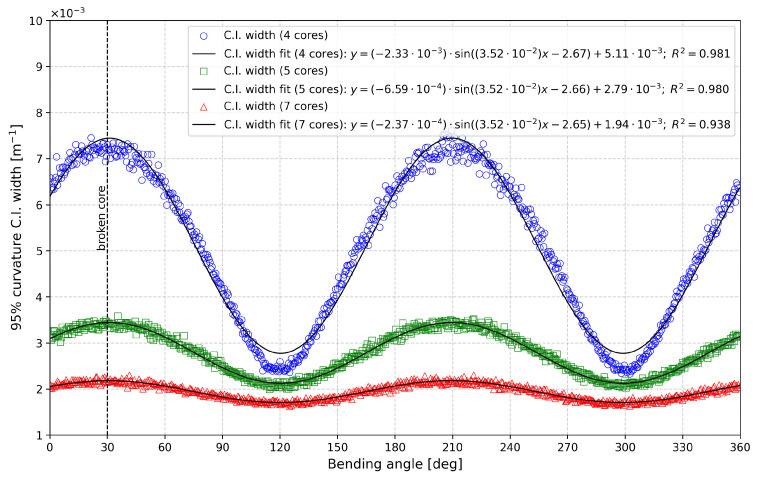
Representation of 95% curvature C.I. width as a function of the bending angle for different numbers of cores in the damaged case.

The results are presented in Figure 8 and Figure 9, which depict the 95% C.I. widths as functions of the bending angle (in degrees) for different numbers of cores in the damaged case. Figure 8 illustrates the bending angle error, while Figure 9 shows the curvature.

In both figures, a vertical dashed black line at 30° marks the location of the failed fiber core. The data in both figures can be modeled using a sinusoidal function of the form $f\left(x\right)=a\sin\left(bx+c\right)+d$, with the fitting equations and corresponding $R^{2}$ values displayed on each graph to evaluate the fit quality.

The first notable difference between the two cases is the phase relationship of the errors. For the bending angle, the 95% C.I. width is minimized at 30° and 210° (30° + 180°) and maximized at 120° (30° + 90°) and 300° (30° + 270°). Conversely, the curvature confidence interval width is maximized at 30° and 210° and minimized at 120° and 300°, indicating a 90° phase difference between the two parameters. This sinusoidal representation also quantifies the benefits of increasing the number of sensing cores. By comparing the *a* parameter (i.e., the amplitude) of the fitted models, we observe that increasing the number of cores from four to five reduces the bending angle error by 72%, while increasing to seven cores yields a 90% error reduction. The same trend is observed for curvature errors, with reductions of 72% and 90% for five and seven cores, respectively, compared to the four-core configuration.

It is important to note that the results we obtained, especially in Figure 8 and Figure 9, show a clear sine-like behavior that is consistent across all 720 bending angles, indicating that 1000 Monte Carlo iterations were sufficient for reliable results. An increased number of Monte Carlo iterations would reduce all confidence interval values and slightly improve curve quality (i.e., less point-by-point variation), but the trend is already clearly visible.

### 3.3. Effect of Core Failure on Curve Reconstruction

**Figure 10:**
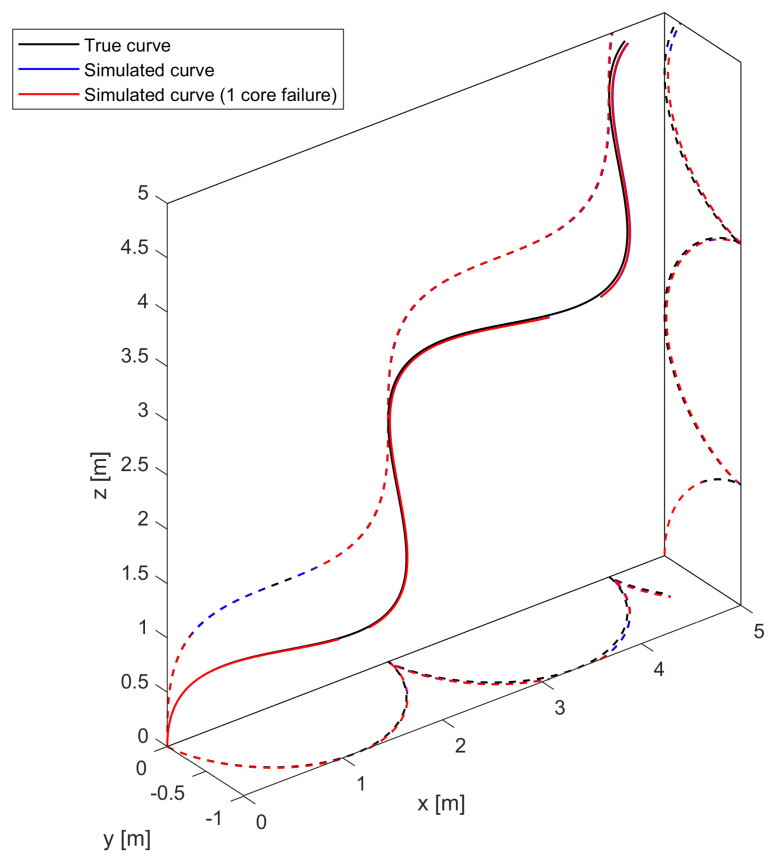
Three-dimensional plot of right-handed helix with its orthogonal projections.

Up to this point, we have focused on the effect of the loss of a sensing core on the measurement of the curvature vector in a single sensing point. In this section, we focus on how core failure affects the reconstruction of an entire 3D curve. First, using the process described in Section 2.3.2, we generate a right-handed helix curve (defined by a constant curvature of κ= 1 m^−1^ and a torsion of τ= 1 rad/m) with an arbitrary length of 10 m and 1000 sensing points to better control the evolution of the error. Monte Carlo simulations for sensing cables with a variable number of sensing cores, both pristine and with one missing core, are performed with 10,000 iterations. The shape obtained in one Monte Carlo iteration is shown in Figure 10 for illustrative purposes.

**Figure 11:**
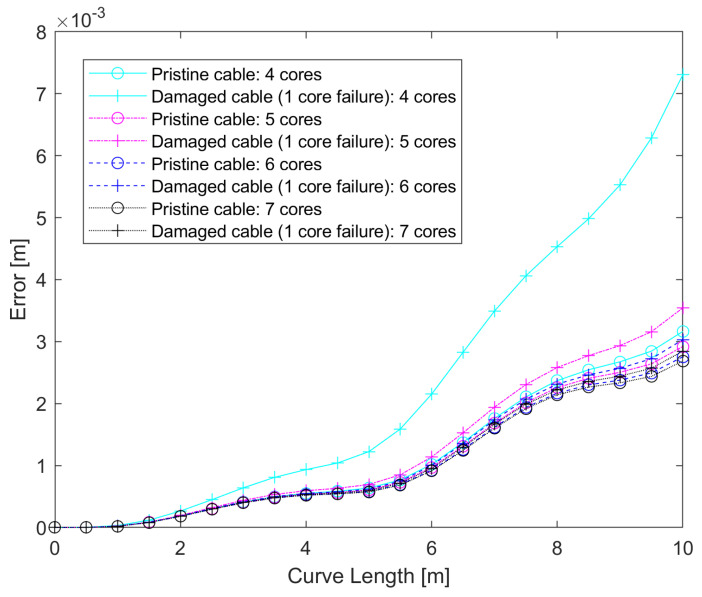
Error trend comparison for 4, 5, and 7 cores: pristine vs. 1 core failure.

Figure 11 shows the average of the errors at every point of the curve for all iterations, which shows that the error tends to increase along the curve as the effect of noise is compounded, as expected, and that the differences between pristine and damaged cables tend to increase, but that this effect quickly decreases as the number of cores is reduced. Table 2 shows the highest error (found at the final point of the curve) for pristine and damaged cables, as well as the ratio between the two. The error ratio decreases by 46.9% as the number of cores increases from four to five cores. However, the improvement becomes less evident as the number of cores increases, with six and seven cores already showing similar results (only 2.8% of error ratio reduction).

**Figure 12:**
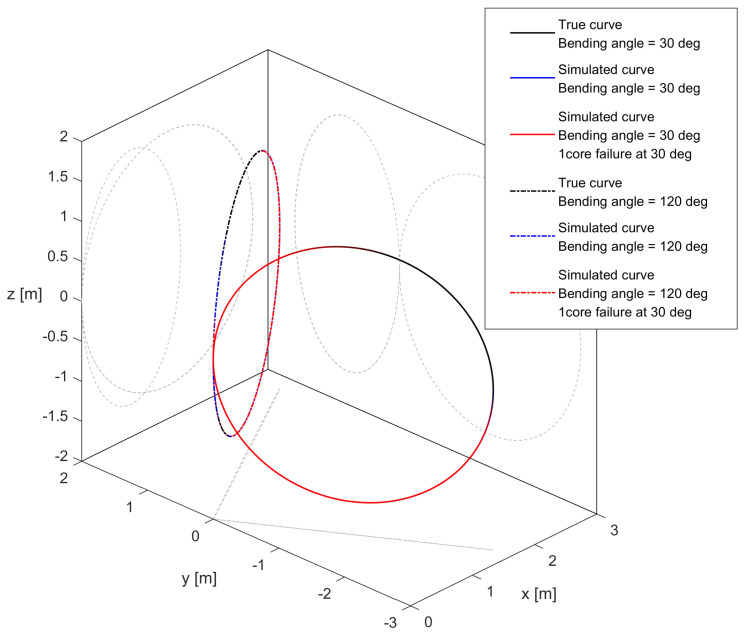
Curve reconstruction for circles (with their orthogonal projections) having $\alpha_{0}=$ 30° and $\alpha_{0}=$ 120° pristine and damaged cable with four sensing cores.

**Figure 13:**
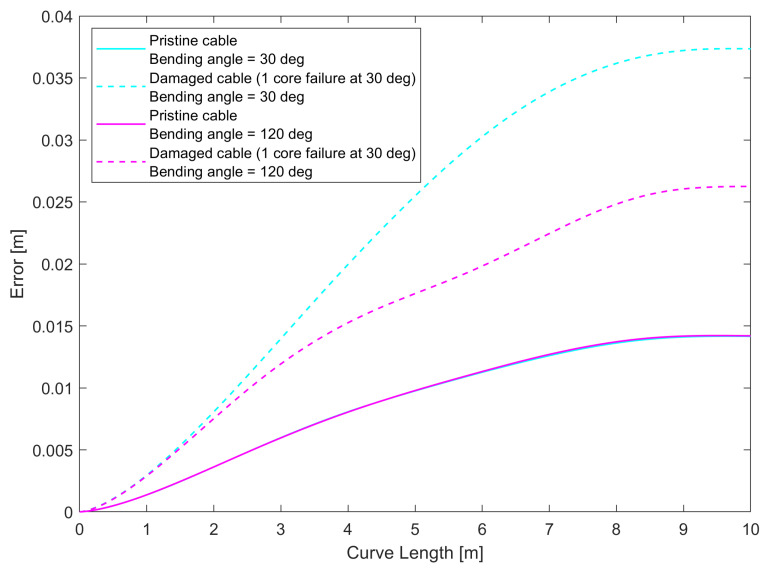
Error curves for circles having $\alpha_{0}=$ 30° and $\alpha_{0}=$ 120° pristine and damaged cable with four sensing cores.

**Figure 14:**
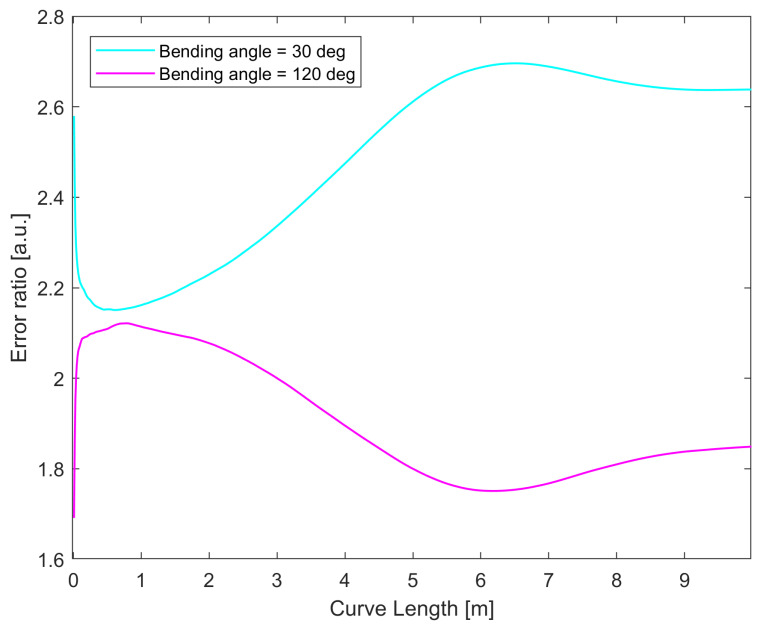
Point-by-point error ratios between the damaged and pristine cables with four sensing cores for circles having $\alpha_{0}=$ 30° and $\alpha_{0}=$ 120°.

While the helix simulation provides some insight, the curve does not allow us to see the effect of asymmetry in noise measurement for curvature and bending angle in the presence of core breakage, since the bending angle changes constantly along the curve, due to the non-zero torsion. As a further test, we chose to perform the simulation for curves lying on a plane, such as those shown in Figure 12: we simulated shape sensing for a pristine and damaged cable along two different circles (a shape with a constant curvature and bending angle), where the bending angle was equal to 30° (that is, the curvature vector passes along the damaged core), or 120° (that is, the curvature vector is perpendicular to the axis containing the damaged core). As shown in the previous section, for 30°, the error reached the maximum for the curvature and minimum for the bending angle, and vice versa for 120°. We simulated the 3D reconstruction following the procedure described in Section 2.3.2 for the four following situations: circle at 30° using an intact cable, circle at 30° using a cable with a broken core, and the same two cases for 120°. The errors along the curve length are shown in Figure 13, and the error ratios between the damaged and pristine cable for the two curves are shown in Figure 14. What can be seen from this last figure is that the noise increase is more stark when the curvature vector is oriented in such a way that the noise of the curvature is at its maximum.

The results displayed were obtained with a four-core sensing cable, but similar results were found with sensing cables with higher numbers of cores as well, although the noise ratios declined with higher numbers of cores, similar to what was found for the helix, with the biggest gains obtained when going from four to five cores. One thing to note is that in Figure 14, the error ratio does not increase along the length of the curve when the bending angle noise is at its maximum. This can be linked to the fact that circles are closed curves, so the evolution of the errors is different compared to a curve that does not return to its origin, like a helix. These results suggest that when the reconstructed curve is on a plane, the loss of a sensing core will mean that the accuracy of the measurement will depend on the orientation of this plane with respect to the angular position of the broken core. In particular, the loss of performance is minimized when the plane is perpendicular to the line connecting the broken core to the center of the cable, and maximized when it is parallel to it.

## 4. Conclusions

In this work, we have investigated how the loss of a sensing core affects the uncertainty of shape sensing performance, both in terms of the measurement of the curvature vector in a single sensing point and overall reconstruction of the 3D shape. In terms of the single sensing point in the sensing cable, we have confirmed a linear relationship between measurement noise and uncertainty in curvature and bending angle. We also found that the loss of a sensing core affected the uncertainty for both curvature and bending angle by a constant factor independent of the number of cores. In addition, we characterized how the asymmetry introduced by the loss of a sensing core also affected the measurement of the curvature vector, with polar analysis showing that, in this situation, the measurement accuracy depended on the orientation of the vector itself, equal to the bending angle. In particular, if the polar coordinates of the failed core were (r,θ), the noise of the curvature magnitude was found to be minimized when the bending angle was equal to $\theta +\frac{\pi }{2}+n\pi$ for any integer *n*, and maximized when the bending angle was equal to θ+nπ. For the bending angle, it was the opposite, with the noise being maximized for angles of $\theta +\frac{\pi }{2}+n\pi$ and minimized for θ+nπ. This asymmetry in measurement performance is believed to be linked to the varying amount of information a single core can hold for the two measurements: cores along the curvature vector direction provide more information on the curvature magnitude, while cores on the neutral axis provide more information on the bending angle. For both curvature and bending angle, the change in measured noise as a function of the bending angle was found to follow a sine relationship, and an increase in the number of cores reduced this error value by 72% going from four to five cores, and by 90% going from four to seven cores.

Regarding overall 3D reconstruction, we first investigated a situation where the orientation of the curvature vector varied continuously, taking a helix as an example, where we saw that the error increase factor caused by core breakage grew progressively along the curve, and was reduced by increasing the number of cores, with the greatest reduction occurring by raising the number of cores from four to five, which is in accordance with the results obtained for the single sensing point, by reducing the increase in uncertainty due to the moving bending angle. Finally, by simulating circles resting on different planes, it was possible to see the different impact that increased noise on either curvature or bending angle can have on shape reconstruction, and it was found that increased noise in curvature had the largest impact. When the shape being measured mostly lay on a single plane (meaning the bending angle was mostly constant) and a single core at polar coordinates (r,θ) was lost, the performance was best when the bending angle was equal to $\theta +\frac{\pi }{2}+n\pi$ and worst when equal to θ+nπ. This result indicates that the orientation of the plane the shape lies on is an important factor when the breaking of one of the sensing cores can occur and that the overall noise on the curvature seems to have a slightly greater effect on 3D reconstruction compared to noise affecting the bending angle.

What we can take away from these results is the following:

- The asymmetry introduced by the loss of a sensing core causes the uncertainty in both curvature and bending angle to depend on the orientation of the curvature vector, with their maximum and minimum values occurring at opposite angles.
- The effect of this asymmetry on measurement uncertainty is reduced by an increased number of sensing cores, with the highest improvement seen when going from four to five cores.
- The accuracy of 3D reconstruction for shapes that mostly lie on a plane will depend on the plane’s orientation with respect to the missing core.

These results are significant in highlighting the potential and consequences of performing shape sensing even when a core experiences failure, a scenario that is plausible in many industrial applications utilizing fiber bundles. In addition, they not only suggest best practices for improving measurement performance in the event of core breakage, but also highlight critical aspects to take into account when assessing how accuracy can change in the presence of core breakage, including the nature of the shape being investigated. In industrial settings, these findings might prove helpful in the following ways:

- If it is established that the possibility of core breakage is likely (for instance, if the insertion or removal of cables is difficult and potentially damaging), then a suitable sensing cable configuration can be chosen to allow shape sensing with an acceptable accuracy even if one of the sensing cores is lost. While, in general, adding more sensing cores will provide better results, each additional core corresponds to an increased length of fiber that has to be monitored, with consequent costs, either in terms of measurement range, measurement bandwidth, or measurement channels, depending on the type of optical fiber sensor employed. The results in this paper suggest that a sensing cable with four peripheral and one central core provides the best compromise in terms of retained accuracy with the minimum number of cores. In the case where higher accuracy is required, additional sensing cores can be added, and we estimated the improvement for multiple configurations.
- When core breakage occurs, an industrial user might have the need to decide whether the cable can still be used or it has to be replaced. While replacement would be the optimal solution for maintaining sensing performance, it might be a difficult process depending on the application. Since in industrial environments, the goal of a sensor is usually to determine a specific set of events with a certain degree of reliability (that is, a minimum probability of detection), it would be worthwhile to determine whether a damaged cable is still viable for the required specifications. In this context, the results presented in this paper show a way to determine how the accuracy of the cable can change in the presence of core breakage and specific aspects that should be taken into consideration, such as the orientation of the broken cable with respect to the bending angles that are detected, which might also have an impact on measurement accuracy, especially if the shape that is being detected mostly lies on a specific plane, which can be expected to occur in some applications (e.g., shape sensing for railway monitoring). All of these factors that we highlighted in this work could have a significant impact on the decision-making process for deciding whether the sensing cable has to be replaced or can keep being employed.

As an additional consideration, the results presented in this work can be important even for the potential development of dynamic approaches to mitigate the effect of the loss of a sensing core, such as the employment of Kalman filters [29]. In order for these dynamic filtering approaches to function, it is important for the overall standard deviation of the measurement to be known in advance, including its dependency on other factors in the measurement, such as the bending angle and the location of the damaged core.

In future works, we intend to implement experimental tests to confirm the results presented here. In this paper, we focused on numerical simulation for mainly two reasons. First, obtaining a sufficient amount of data for reliable accuracy statistics would require a large number of measurements for each specific condition, which is impractical, especially for the larger-scale nature of shape sensing for SHM applications. Second, these simulations were intended to be independent of the specific optical fiber sensor employed to gather data. In an actual experiment, the specific sensing technique (BOTDA, ϕ-OTDR, FBG array) would influence overall measurement accuracy and would require dedicated investigation.

Especially for the latter reason, an experimental evaluation would be valuable to expand the results by taking into consideration other aspects that are difficult to simulate. Beyond the specific effect of the sensing method, other examples of aspects that could be taken into consideration include the consequences of slippage for fiber bundles, where bending causes the displacement of sensing cores with respect to their expected position, as well as evaluating the impact of such displacements.

Another aspect, particularly relevant in cases where all sensing cores need to be connected sequentially (as in the case of BOTDA), is the fact that the sensor acquires a single strain trace that contains the information of each sensing core in different segments, and the determination of their position also represents an additional uncertainty contribution.

These are all examples of aspects of the sensing process that can affect overall performance, which need to be studied to provide better insights for both cable design and the decision-making process in shape sensing applications.

## Figures and Tables

**Table 1:** Simulation parameters.

Parameter	Symbols	Values	Units
Core offset	$r_{c}$	2.1	[mm]
Measurement noise standard deviation	$\sigma_{\varepsilon }$	50.0	[με]
Target curvature	κ	10.0	[m^−1^]
Longitudinal strain	$\varepsilon_{long}$	0.0	[με]
Number of tested directions	$N_{\alpha }$	720	[−]
Monte Carlo iterations	*N*	10^3^	[−]

**Table 2:** Error analysis.

N.	Error	Error	Error Ratio	Error Ratio Reduction
Cores	Pristine Cable	One Core Failure Cable	(Last Point)	(One Core Increment)
4	3.2 mm	7.3 mm	2.28	-
5	2.9 mm	3.5 mm	1.21	46.9%
6	2.8 mm	3.0 mm	1.07	11.6%
7	2.7 mm	2.8 mm	1.04	2.8%

## Data Availability

The raw data supporting the conclusions of this article will be made available by the authors on request.

## Abbreviations

The following abbreviations are used in this manuscript:

C.I.	Confidence Intervals
FBG	Fiber Bragg Grating
OFSs	Optical Fiber Sensors
SHM	Structural Health Monitoring
SLAM-DAST	Smart LightwAve Multi-modal Distributed Acoustic Strain and Temperature sensor
SSE	Sum of Squared Errors
